# Getting to the Heart of the Matter: Myocardial Injury, Coagulopathy, and Other Potential Cardiovascular Implications of COVID-19

**DOI:** 10.1155/2021/6693895

**Published:** 2021-04-22

**Authors:** Aaron Schmid, Marija Petrovic, Kavya Akella, Anisha Pareddy, Sumathilatha Sakthi Velavan

**Affiliations:** Division of Biomedical Sciences, Marian University College of Osteopathic Medicine, Indianapolis, USA

## Abstract

COVID-19 was primarily identified as a respiratory illness, but reports of patients presenting initially with cardiovascular complaints are rapidly emerging. Many patients also develop cardiovascular complications during and after COVID-19 infection. Underlying cardiovascular disease increases the severity of COVID-19 infection; however, it is unclear if COVID-19 increases the risk of or causes cardiovascular complications in patients without preexisting cardiovascular disease. The review is aimed at informing the primary care physicians of the potential cardiovascular complications, especially in patients without underlying cardiovascular disease. A comprehensive literature review was performed on cardiac and vascular complications of COVID-19. The primary cardiac and vascular complications include myocarditis, acute coronary syndrome, myocardial injury, arrhythmia, heart failure, shock, multisystem inflammatory syndrome, venous and arterial thrombotic events, stroke, and coagulopathy. A detailed analysis of the pathogenesis revealed six possible mechanisms: direct cardiac damage, hypoxia-induced injury, inflammation, a dysfunctional endothelial response, coagulopathy, and the catecholamine stress response. Autopsy reports from studies show cardiomegaly, hypertrophy, ventricular dilation, infarction, and fibrosis. A wide range of cardiac and vascular complications should be considered when treating patients with confirmed or suspected COVID-19 infection. Elevated troponin and natriuretic peptides indicate an early cardiac involvement in COVID-19. Continuous monitoring of coagulation by measuring serum D-dimer can potentially prevent vascular complications. A long-term screening protocol to follow-up the patients in the primary care settings is needed to follow-up with the patients who recovered from COVID cardiovascular complications.

## 1. Introduction

The World Health Organization (WHO) describes coronaviruses as a group of viruses, several of which infect humans, which usually cause respiratory disease or illness, ranging from the common cold to severe acute respiratory distress syndrome (ARDS) [1]. The most recently discovered coronavirus, SARS-CoV-2, causes the infectious disease COVID-19, a pandemic affecting many countries around the world [1]. COVID-19 typically presents clinically as fever, dry cough, dyspnea, and fatigue, but signs and symptoms vary widely [1]. Reports are emerging of patients presenting with cardiovascular symptoms, including chest tightness and heart palpitations [2, 3]. Some patients present exclusively with cardiac complaints and do not have any other respiratory complaints [3]. Underlying cardiovascular disease increases the risk of severe COVID-19 disease and death [4, 5], but does COVID-19 infection increase the risk of, or cause, cardiovascular complications or cardiovascular disease in patients without underlying cardiovascular disease?

Dweck et al. (2020) demonstrated that the cardiovascular system seems to be one of the most common organ systems affected by COVID-19. After excluding patients with preexisting ischemic heart disease, heart failure, and valvular heart disease, 46% of patients with COVID-19 with an indication for echocardiography (suspected left ventricular failure, suspected right heart failure, and elevated cardiac biomarkers) had echocardiographic abnormalities including myocardial infarction, myocarditis, and Takotsubo cardiomyopathy [6]. Another study showed 50% of COVID-19 patients had electrocardiographic abnormalities, and 42% experienced chest pain, palpitations, or dyspnea [7]. In the same study, 75% of cases in the same study had cardiac magnetic resonance (CMR) abnormalities [7]. These complications are consistent with previous case reports [8–10]. COVID-19 triggers several cardiac sequelae, including myocardial injury, acute coronary syndromes, myocarditis, arrhythmias, and acute onset heart failure [11–14]. The overall incidence of cardiac injury caused by COVID-19 varies from 7-64%, depending on the parameter used, among hospitalized patients in various reports [6, 7, 11–13].

COVID-19 can also manifest as vasculopathy. Recent studies have shown the impact that COVID-19 has had on increasing the risk of coagulopathies, including thrombotic events such as deep venous thrombosis (DVT), coagulopathy, and stroke [15–18]. General risks for coagulopathy and pathogenesis of thrombosis include age, acute illness, being bedridden, stasis, genetics, fever, diarrhea, sepsis, liver injury, CKD, COPD, HF, and malignancy [19].

This review intends to provide the primary care physicians with concise knowledge of potential cardiovascular complications in COVID-19 patients to understand the disease course better and decide how to manage and treat patients. Major common cardiac and vascular complications in COVID-19 patients were investigated, with an emphasis on those without known underlying cardiovascular disease. The authors expect this review will contribute to the intake and management of patients with suspected and confirmed COVID-19 infection making evaluation and treatment safer and more efficient. The aim is to ensure physicians are up to date on the cardiovascular complications that can arise from COVID-19.

## 2. Main Text

### 2.1. Pathogenesis of COVID-19 Cardiovascular Disease

The exact mechanism by which COVID-19 causes myocardial injury and damage is being investigated. Kim et al. proposed six different mechanisms by which COVID-19 infection may cause cardiovascular injury and manifestations [20]. These mechanisms do not individually explain the cardiac injury caused by COVID-19, but rather multiple factors affect the cardiovascular system in different ways.

1. *Direct Cardiac Damage Mediated via an Angiotensin-Converting Enzyme (ACE-2) Receptor-Dependent Myocardial Infection* [20]. ACE-2 receptors are expressed in the heart, more highly expressed than in the lungs [21], and the SARS-CoV-2 virus binds and utilizes these receptors. Also, this virus can decrease angiotensin 1-7 levels, which play a protective role against cardiovascular complications [22]
2. *Hypoxia-Induced Injury from Oxidative Stress, Acidosis, and Mitochondrial Damage* [20]. Researchers built on the idea of hypoxia-induced cardiac injury stating that the common manifestation of respiratory distress caused by COVID-19 infection can result in hypoxemia leading to an oxygen supply and demand mismatch [23, 24]. Hypoxemia arising from COVID-19-induced lung injury may lead to injury of cardiomyocytes as hypoxemia can cause accumulation of metabolites, including oxygen free radicals [25]
3. *Inflammation Leading to Vessel Hyperpermeability and Angiospasm, Causing Microvascular Damage to the Heart* [20]. A dysfunctional endothelial response during COVID-19 infection, due to direct COVID-19 infection of endothelial cells, aging, or chronic disease, turns into increased apoptosis and disruption of intercellular junctions. This, in turn, leads to increased permeability, leakage of fluid, leukocytes, and proteins. This hyperpermeability and leakage then interfere with oxygen exchange in the lungs and induce microcirculatory disorders in the heart [26]
4. *Damage Mediated by a Systemic Inflammatory Reaction and Cytokine Storm* [20]. Research suggests that in COVID-19-infected patients, the systemic inflammatory reaction, immunologic derangement, and cytokine storm can lead to arrhythmia, thrombosis, coagulopathy, and other cardiovascular sequelae [23]. Guo et al. showed that rising troponin levels, indicating myocardial injury, in patients with COVID-19 correlated to rising C-reactive protein levels suggesting that the myocardial injury may be inflammation-mediated or related to an inflammatory mechanism. Inflammatory cytokines released due to COVID-19 infection may lead to tissue damage and dysfunction, reduce coronary blood flow and oxygen supply, and may lead to plaque destabilization and the formation of microthrombi [27]. Studies have shown that severe COVID-19 patients are exposed to high concentrations of cytokines [28]. Release of cytokines and damage-associated molecular patterns trigger endothelial activation leading to vasodilation and prothrombotic events [19, 29, 30]. These aspects of COVID-19 infection are driving factors of ARDS, coagulopathy, and COVID-19-related cardiovascular complications [31]
5. *Vessel Occlusion as a Result of Coagulopathy, Thrombosis, Embolus, Plaque Instability, or Plaque Rupture from the Systemic Inflammatory Response* [20]. Occlusion leads to myocardial ischemia and infarction. COVID-19 patients are prone to venous thrombosis due to fever and diarrhea, hypotension due to dehydration, secondary infections, and prolonged bed rest [17]. Direct infection of endothelial cells increases cytokine production, enhances tissue factor expression, and increases NF-*κ*B [32]. This, in turn, can trigger an inflammatory response and activation of the coagulation pathway, creating an ideal environment for thrombotic events to occur [32, 33]. Platelets from critically ill COVID-19 patients showed upregulation of eIF4E, p38, and phosphorylation of ERK1/2, which indicates increased activation of the MAPK pathway. Increased MAPK signaling promotes thromboxane generation, a mechanism triggering platelet hyperactivity that may be present in COVID-19 patients [34]. Thrombotic events in patients with COVID-19 may be due to antiphospholipid antibodies. Still, it is difficult to differentiate the cause of a thrombotic event as it may coincide with other causes such as DIC, heparin-induced thrombocytopenia, and thrombotic microangiopathy [35]
6. *Another Possible Cause Is Stress-Induced Cardiomyopathy and Cardiac Injury* [20]. Increased cardiac workload, up to eight-fold, during viral infection, to compensate for the increased metabolic demands, compounded by the many effects of the infection on the lungs and breathing, considerably impairs cardiac function [36]

To summarize, direct injury, hypoxia, microvascular damage, and systemic inflammatory syndrome may all lead to inflammation of the heart, myocarditis, and acute cardiac injury.

### 2.2. Cardiac Complications

Myocarditis and acute cardiac injury further complicate the infection with the potential to lead to arrhythmia, heart failure, and cardiogenic shock [20]. Pertinent cardiac findings from several COVID-19 studies are presented in Table 1.

#### 2.2.1. Myocarditis

Myocarditis has many different causes, including infection (viral, bacterial, etc.), autoimmune disease (SLE, etc.), and medications. Myocarditis can also be idiopathic. Inflammation seen in myocarditis can be focal or global, can lead to chamber dysfunction or necrosis, and has the potential to cause chronic cardiovascular complications [37].

The clinical presentation of COVID-19 is like the myocarditis of any etiology and includes fever, hypotension, dyspnea, chest pain, and arrhythmia. Viral myocarditis may also mimic myocardial infarction, acute coronary syndrome, or heart failure with nonspecific electrocardiographic changes (ST elevation, T-wave inversion, ST depression, and pathologic Q-waves), elevated enzymes, hemodynamic instability, tachycardia, displaced point of maximal impulse, or S3/S4 gallop. Myocarditis may also progress to heart block, arrhythmia, and impairment of left ventricular function [37–41].

Myocarditis, of any cause, is often preceded by flu-like and gastrointestinal symptoms. These are also some of the most common presenting symptoms of patients with COVID-19, making the diagnosis and management of the disease process tricky [42]. COVID-19-related myocarditis manifestation can range from mild symptoms to acute-onset heart failure. Patients who present with fulminant myocarditis often have a fever, sinus tachycardia, cold extremities, and low pulse pressure [43]. Recognition that viral myocarditis with the frequent rise in troponin is integral to the diagnostic and treatment approach considered in COVID-19-infected patients.

Fulminant myocarditis and heart failure have also occurred in COVID-19-infected patients [44]. Multiple case reports of fulminant myocarditis in COVID19 patients without underlying cardiovascular disease have been described [9, 19, 45]. While this type of injury could be due to direct damage to the heart by circulating virus, it is suspected to be mediated by immune system reaction and inflammation as most cases of fulminant myocarditis were sporadic and resolved after treatment [46]. An occurrence of acute myocarditis in an adolescent patient was reported. The patient had almost none of the typical signs and symptoms except fever, thus, indicating testing for COVID-19 in the pediatric age group with features suggestive of acute myocarditis [47].

A study of 1,216 patients with COVID-19 infection showed that 55% of patients that received an echocardiogram (due to suspected left or right heart failure, chest pain with ST-elevation, cardiac biomarker elevation, ventricular arrhythmia, suspected tamponade, or cardiogenic shock) had an abnormal echocardiogram. Left ventricular abnormalities were seen in 39%, and right ventricular abnormalities were seen in 33%. Evidence for myocardial infarction was seen in 3%, and evidence of myocarditis was seen in 3%. Of the 1216 patients, 901 (74%) patients did not have an underlying cardiac disease; still, 46% of them had abnormal echocardiograms, and 13% had severe disease. 25% of those without the preexisting cardiac disease had abnormal left ventricles, 33% had abnormal right ventricles [6].

#### 2.2.2. Acute Coronary Syndrome (ACS)

Increased risk for thrombotic events can lead to increased ACS risk in COVID-19 positive patients. Furthermore, upon onset of inflammation, ACS can be caused by plaque rupture due to macrophage activation, endothelial cell activation, smooth muscle cell activation, tissue factor expression, and further inflammation onset due to platelet activation [20]. Cases of both occluded and patent myocardial infarctions have been observed previously in the setting of viral illnesses [48–50].

Although the frequency remains unclear, myocardial injury with ST-segment elevation has been observed in COVID-19 patients. In a case series of 18 patients with COVID-19, 14 had focal ST-segment elevation, and four had diffuse ST-segment elevation. Ten patients had ST-segment elevation upon presentation. The other eight patients developed ST-segment elevation during hospitalization. Still, their treatment is unknown, and if it included azithromycin or hydroxychloroquine, it might have contributed to the development of rhythm abnormalities. Eight patients had a reduced left ventricular ejection fraction [8]. Several other case reports have demonstrated ST-segment elevation or Brugada pattern on electrocardiography [51–54]. The link between fever and Brugada pattern and cardiac arrest has been described extensively in the literature. Patients with Brugada pattern and COVID-19 should be monitored regardless of their respiratory conditions [55].

It is essential to note the overlapping disease course and symptomatology between ACS and COVID-19 [12]. The signs and symptoms of infection and cardiac damage can present similar to acute coronary syndrome; therefore, a high index of suspicion for differential diagnoses must be required by physicians, and alternative noninvasive diagnostic methods utilized [20].

#### 2.2.3. Biomarkers of Myocardial Injury

The definition of myocardial or cardiac injury varies between studies, and no integrated definition is currently present. Varying definitions of myocardial injury are presented in Table 2.

Elevated cardiac biomarker levels, myocardial inflammation, electrocardiographic abnormalities, and echocardiographic abnormalities are highly prevalent in patients with COVID-19. These signs are associated with a more severe disease course and a worse prognosis. Signs of myocardial injury have been seen in up to 30% of hospitalized COVID-19 patients [4, 27, 56, 57]. Evidence of myocardial injury is most often correlated to elevated troponin levels. Potential causes for elevated troponin in COVID-19 patients include myocarditis, cardiomyopathy, hypoxic injury, ischemic injury (increased oxygen demand due to fever or tachycardia, or decreased oxygen supply due to hypotension or hypoxemia), vascular or microvascular damage (due to increased reactive oxygen species, endothelin imbalance, or endothelial dysfunction), and systemic inflammation (cytokine storm). The clinical value of troponin levels outside of acute coronary syndrome, myocardial infarction, and heart failure is difficult to quantify [27]. While abnormal troponin levels may lead to unnecessary use of resources and testing, all action must be taken to appropriately monitor and treat COVID-19-infected patients due to the similar presentation of ACS and COVID-19 infection and the potential for fatal outcomes.

Further research is needed to establish the clinical value of troponin levels in COVID-19-infected patients. Elevated troponin levels were associated with elevated levels of CRP and NT-proBNP, linking myocardial injury to the severity of inflammation and ventricular dysfunction [27].

#### 2.2.4. Arrhythmia

Arrhythmias represent one of the complications of COVID-19 infection, demonstrated in 16.7% of patients with an increased prevalence of 44.4% in patients admitted to the ICU [4, 58]. Arrhythmia was demonstrated in another study in 16.7% of hospitalized COVID-19 patients [13]. There appear to be more possible mechanisms causing arrhythmia in COVID-19 patients than other cardiac complications, possibly explaining its high prevalence. Arrhythmia could result from myocarditis, myocardial ischemia, infection-induced hypoxia, fever, metabolic disarray, hormonal dysregulation, medication, or inflammation.

Myocarditis-induced arrhythmias have been seen in COVID-19 positive patients in an acute setting or chronic myocarditis [43]. Electrolyte abnormalities, in particular, hypokalemia, which can occur in any systemic illness, may also be of particular concern in causing arrhythmia in COVID-19-infected patients due to the virus' interaction with the renin-angiotensin-aldosterone system [59]. Myocarditis or myocardial inflammation could result in sinus node dysfunction and, ultimately, arrhythmia in COVID-19 patients [60, 61].

Palpitations were one of the most common presenting symptoms in 7.3% of patients in a cohort of 137 COVID-19 patients [11]. More serious arrhythmia complications shown to arise in COVID-19 infection include ventricular tachycardia, atrial fibrillation, ventricular fibrillation, atrioventricular block, and cardiogenic shock [4, 58, 62]. Patients diagnosed with COVID-19 infection that have elevated cardiac biomarkers may develop new-onset arrhythmia. New-onset arrhythmia in the setting of elevated cardiac biomarkers can indicate myocarditis and should raise suspicion for underlying myocarditis. Primary care physicians must include this in their treatment plan and provide adequate monitoring and follow-up [4].

The risk of arrhythmia could also be associated with the usage of early COVID-19 treatments such as azithromycin and hydroxychloroquine. The use of hydroxychloroquine and azithromycin has independently been shown to cause arrhythmia [63, 64]. While they were used in COVID-19 patients, no studies were found separating COVID-19 patients experiencing arrhythmia into groups receiving the drug(s) and not receiving the drug(s). This makes it even more challenging to discern a cause of arrhythmia and the true prevalence of arrhythmia in patients with COVID-19 infection not taking hydroxychloroquine or azithromycin [11–13, 65]. Another evidence showed that many patients might have higher QT values even before starting the drugs potentially causing further QT prolongation [66]. In patients grouped under “Out of hospital arrests” during the COVID-19 pandemic, a study on post-ROSC (return of spontaneous circulation) showed prolonged QTc values in 12 out of 27 patients [67]. The incidence of arrhythmia needs to be further evaluated in future studies in COVID-19 patients not given azithromycin or hydroxychloroquine, as the exact contribution of COVID-19 infection to arrhythmia development is unknown. Hence, physicians must keep the risk of arrhythmia and myocarditis in mind when treating and evaluating COVID-19 patients.

#### 2.2.5. Out of Hospital Cardiac Arrest (OHCA)

A higher incidence of out-of-hospital cardiac arrest has been noted in patients suspected of or with a confirmed diagnosis of COVID-19 [68] [67]. Even in patients on whom emergency personnel attempted resuscitation, OHSA occurred at 14.9% points more in 2020 as compared to 2019 [69]. Another study observed a lower attitude of laypeople in initiating cardiopulmonary resuscitation during the COVID-19 outbreak compared to 2019. The study also noted that a confirmed or suspected COVID-19 infection did not influence the resuscitative attempts by BLS and ALS staff [70]. A doubling of the incidence of OHCA and a simultaneous reduction in survival rate that was noticed during the COVID-19 pandemic was also noted in Paris. The rise was partly attributed to COVID-19 infection and indirectly related to lockdown and health care services-related adjustments [71]. When the number of COVID-19 cases peaked in New York City, a three-fold higher number of OHCA underwent resuscitation compared to the same period in 2019 and most were older, more likely to have specific comorbidities [72]. A study across a longer time period confirmed that the OHCA trend paralleled the ascending and descending phases of the COVID-19 pandemic [73].

#### 2.2.6. Heart Failure

Heart failure has been shown to occur in up to 23% of infected patients. They seem to occur secondary to exacerbation of left ventricular dysfunction, myocarditis, acute coronary syndrome, arrhythmia, pulmonary hypertension, ARDS, or cardiomyopathy [12, 74]. The incidence of cardiomyopathy in critically ill ICU patients has yet to be evaluated on a large scale but has been shown to develop in up to 33% of patients [75]. A small case series of COVID-19 patients with acute cor pulmonale showed profound hemodynamic instability and cardiac arrest with acute right ventricular failure [76].

#### 2.2.7. Children and Multisystem Inflammatory Syndrome

The pediatric population appear to have a milder clinical course but may still show some cardiac complications such as arrhythmia and must be monitored [77]. A systemic inflammation syndrome, like Kawasaki disease, has been shown in children as well. A case report of a pediatric patient diagnosed with COVID19, presenting with Kawasaki-like disease, showed arrhythmia, electrocardiographic abnormalities, elevated biomarkers, left ventricular systolic dysfunction, pericardial effusion, delayed capillary refill, and gallop on auscultation [78]. Kawasaki disease is associated with left ventricular systolic dysfunction in 20% of patients, coronary artery dilation in 29%, and mitral regurgitation in 27%, suggesting shared mechanisms between Kawasaki disease, cardiac complications and abnormalities, and myocarditis [79]. Despite having a milder clinical course, children should be monitored for cardiac complications and, upon presenting with any Kawasaki-like disease, should be tested for COVID-19.

### 2.3. Vascular Complications

A wide range of vascular complications has been recognized in patients affected with COVID-19 that include venous and arterial thrombosis, neurological ischemic events, and coagulopathy as summarized in Table 3.

#### 2.3.1. Venous Thrombotic Events (VTE)

In a recent study, 184 COVID-19 positive patients in the ICU were evaluated for thrombotic events. All the patients received standard doses of thromboprophylaxis following hospital protocol upon arrival [80]. The patients were in the hospital for a median duration of 7 days: 27% of the patients had confirmed VTE by CTAP or ultrasonography and 3.7% had arterial thrombotic events [80]. Approximately 31% of patients with COVID-19 in the ICU had thrombotic complications despite thromboprophylaxis [80]. These patients also exhibited prolonged PT (prothrombin time) and aPTT (activated partial thromboplastin time) time [80].

#### 2.3.2. Concomitant Venous and Arterial Thrombotic Events

In a case study, a COVID-19 positive patient was observed to have a severe case of venous thrombosis and arteriosclerosis obliterans of the lower extremities. The patient had a past medical history of uncontrolled type 2 diabetes. Upon arrival to the hospital, lab report showed marked C-reactive protein increase and elevated D-dimer level of over eight ug/mL with a normal PT and aPTT. Vascular ultrasound found DVT in the left lower extremity (LLE) and dorsalis pedis artery occlusion in the LLE on arrival. Anticoagulation and other supportive therapy were given, and after three days, vascular ultrasound confirmed bilateral lower extremity thrombosis with arterial tibialis anterior occlusion and dorsalis pedis artery occlusion on both lower extremities. This patient specifically had many risks and predispositions for DVT, however, it should be anticipated that an infection like COVID-19 could encourage an even greater hypercoagulable environment as seen with this patient who after three days developed many more clots [18].

Three of four COVID-19 patients in another case series had dermal arterial thrombosis suggestive of antiphospholipid syndrome, one also developed venous thrombosis, and all four patients had elevated D-dimer [81]. Another case series reported arterial thrombosis in three patients. A patient, one-week post-COVID-19-recovery, presented with thrombotic occlusion of all tibial arteries on the right leg, an aortic thrombus in the visceral aorta, and thrombotic occlusion of the left popliteal artery. A patient with no atherosclerotic or thromboembolism risk had an occlusive thrombus at the aortic bifurcation with occlusion of the right common iliac artery and stenosis of the left common iliac artery. Another patient developed a stroke in the territory of the left middle cerebral artery [82].

Additionally, certain types of rashes in COVID-19 patients may be an early clinical sign of an underlying thrombotic or hypercoagulable state [81, 83]. Primary care physicians caring for COVID-19 patients must be aware of hallmark manifestations of cutaneous thrombosis including livedoid and purpuric rashes and necrotic eruptions, all associated with elevated D-dimer levels [81].

#### 2.3.3. Stroke

Mao et al. (2020) studied 214 patients that tested positive for COVID-19, and of these patients, 36.4% were found to have neurologic symptoms. 41.1% of the patients in this study were severely infected. They had a higher prevalence of nervous system events such as ischemic stroke and cerebral hemorrhage, as well as symptoms of impaired consciousness. They also had high white cell count, high neutrophil count, lower lymphocyte counts, and elevated C-reactive protein (CRP) levels when compared to patients with nonsevere infection [84]. Through this study, the importance of screening severe patients that are older, presenting with comorbidities and fewer typical symptoms of COVID-19, for the potential of stroke or other neurologic symptoms is evident.

In a case study in Wuhan, China, three critically ill patients that tested positive for COVID-19 were observed to have coagulopathies. Imaging showed that all three COVID-19 positive patients developed multiple cerebral infarcts. Patient 3 developed a thrombotic event 18 days from disease onset, while patients 1 and 2 developed thrombotic events 18 and 33 days after disease onset, respectively. All the patients showed leukocytosis, thrombocytopenia, elevated fibrinogen, elevated d dimer, and presence of anticardiolipin IgA antibody and anti-B2 glycoprotein IgA and IgG antibodies [85].

A retrospective study looked at four COVID-19 positive patients that presented with acute stroke. All patients had hypertension. This may suggest the importance of considering comorbidities, especially hypertension, when analyzing patients for risk of mortality due to COVID-19 presenting with stroke. Two of the patients presented with left shift *h*, while the other two had an elevated cardiac troponin T and D-dimer. IL-6 was only taken in patient 4, and it was elevated, C-reactive protein was tested in patient 1, 3, and 4 and was significantly elevated in all [29]. These results are consistent with other case studies discussed in which patients presented with coagulopathies.

### 2.4. Coagulopathy

During the outbreak of COVID-19 in Wuhan, China, abnormal coagulation patterns were recorded by some investigators. One case study investigated 183 patients who were confirmed COVID-19 positive [16]. Of these patients, 71.4% of nonsurvivors and 0.6% of survivors met the criteria of coagulopathy during their hospital stay [16]. An 11.5% mortality was reported in this study. These patients were revealed to have significantly higher D-dimer and fibrin degradation product levels, prolonged PT, and aPTT time compared to those patients that survived [16]. Although the laboratory findings were consistent with the classic presentation of disseminated intravascular coagulation (DIC), COVID-19 patients generally tend to have thrombotic events rather than the bleeding that is often seen in DIC. However, studies have shown the difference between the presentation of COVID-19-associated coagulopathy (CAC) and classic DIC [86].

COVID-19 patients presenting with vasculopathies often had abnormal PT/aPTT, D-dimer, and platelet counts [15, 16, 18, 19, 29, 80, 84, 85, 87, 88]. Ninety-nine patients with COVID-19 were used in a retrospective study to look at clinical outcomes with symptoms and lab values documented [89]. The aPTT was found to be decreased in 16% of patients, while the PT was found to be decreased in 30% of patients [89]. A much smaller percentage of patients have an increase in PT or aPTT [89]. In CAC, PT and aPTT can be prolonged or normal, while in DIC, they are both elevated. Prolonged aPTT/PT levels may also be due to administration of LMWH in the hospital, and aPTT prolongation may be due to the presence of antiphospholipid antibodies [90]. In DIC, fibrinogen levels may be normal to low due to fibrinolysis suppression from the overproduction of plasminogen activator inhibitor 1 (PAI-1), while in CAC, fibrinogen levels are elevated [86]. Platelet counts may be high or low in CAC, while in DIC, they are consistently low [86]. Low platelet counts can be indicative of platelets forming aggregates that may result in a thrombotic event [87]. Antiphospholipid antibodies are present in lab testing of COVID-19 patients that present with thrombotic events [85]. Complement is activated, and antiphospholipid antibodies are present in CAC, while both are negative in DIC [86]. In both DIC and CAC, the primary cause and target of coagulopathy are the macrophage and endothelial cells resulting in microthrombosis, with additional venous thrombosis seen in CAC [86]. Endothelial dysfunction and direct endothelial infection can cause a dysfunctional endothelial response during COVID-19 infection. This further contributes to the pathogenesis of thrombosis, coagulation disorders, and myocardial injury in COVID-19 patients [26]. Elevated D-dimer and increased inflammatory cytokines IL-1*β* and IL-6 are also seen in both [86]. D-dimer elevation may be due to the vascular disease state created in some COVID-19 patients along with potential multisystem organ involvement, low-grade inflammation, and hypercoagulability [30].

Elevated C-reactive protein was also found in some patients presenting with COVID-19 [18, 29, 84, 89]. C-reactive protein typically increases significantly with inflammation [91]. Inflammation plays a significant role in the pathogenesis of clot formation. C-reactive protein has also been found to promote platelet adhesion to the endothelial ceiling, promoting thrombosis [92]. Studies noted this elevated finding, however, did not discuss it in too much detail. Further studies on the correlation of C-reactive protein and thrombotic events in COVID-19 patients would provide a better sense of the importance of using this lab test in the care of COVID-19 patients.

### 2.5. Pathology

Many studies have reported cardiac autopsy findings in COVID-19 patients (Table 3). The gross findings were reported to be cardiomegaly variably involving the chambers such as global hypertrophy, biventricular hypertrophy or left ventricular hypertrophy, and right ventricular dilatation [93–95]. The histopathological examination revealed myocyte hypertrophy, areas of infarction, lymphocytic infiltration within the myocardium with or without necrosis, epicardial mononuclear infiltrates, and lymphocytic endotheliitis [95–99]. A rare finding of an increased number of megakaryocytes in cardiac tissue with fibrin microthrombi was also noted [100].

The presence of SARS CoV-2 RNA was found in the autopsied hearts [96, 98, 101]. Another report showed isolated cells of heart tissue testing positive for viral RNA, not accompanied by viral replication, infection, or inflammation [102]. Endomyocardial biopsy (EMB) in a COVID-19-infected patient showed low-grade myocardial inflammation and viral particles in the myocardium, suggesting either a viremic phase or, alternatively, infected macrophage migration from the lung [103]. Another study showed myocardial inflammation with elevated lymphocytes and macrophages in the absence of tissue necrosis [104]. EMB serves in an accurate diagnosis and provides tissues for the development of specific biomarkers for SARS-CoV-2 myocarditis. However, its use is limited due to contagious spread risk [43].

### 2.6. Further Implications of COVID-19 on Cardiovascular System

#### 2.6.1. Cardiovascular Symptoms as the Primary Clinical Presentation

The National Health Commission of China (NHC) has shown some patients with later confirmed COVID-19 first presented to the doctor because of cardiovascular symptoms such as heart palpitations and chest tightness [3, 11]. A case report of a healthy patient testing positive for COVID-19 reported findings of myopericarditis accompanied by systolic dysfunction. Additionally, NT-proBNP and troponin T levels were elevated, ST elevation was seen on electrocardiography, and thickened walls and diffuse edema were detected on cardiac magnetic resonance imaging. The patient did not show any respiratory involvement during the clinical course. These findings could indicate eventual heart failure onset [105]. A patient with exertional angina presented without fever or respiratory symptoms showed elevated cardiac biomarkers and tested positive for COVID-19 [106]. Another case report was about a patient with persistent chest pressure for two days. The patient denied cough, fever, and dyspnea. Initial ECG showed ST-elevation. The predominant symptoms the patient presented with were cardiac in nature, and there were no signs of infection; however, the patient tested positive for COVID-19 [107].

#### 2.6.2. Cardiovascular Complications in Patients without Underlying Cardiac Disease

The NHC showed that 11.8% of patients without the underlying cardiovascular disease had substantial heart damage with elevated troponin levels and cardiac arrest [3, 11]. Of the 1216 patients in an echocardiography study of COVID-19 patients, 901 (74%) patients did not have underlying cardiac disease, yet still, 46% of them had abnormal echocardiograms, and 13% had severe disease. 25% of those without preexisting cardiac disease had abnormal left ventricles, and 33% had abnormal right ventricles [6]. Multiple case reports of fulminant myocarditis, cardiac hypertrophy, electrocardiographic abnormalities, echocardiographic abnormalities, elevated biomarkers, and shock have also all been described [9, 45]. A patient without any past medical history was detailed, showcasing the development of atrial fibrillation days after the onset of COVID-19 infection [62]. Another patient with COVID-19 presented with T-wave inversion, elevated troponin and natriuretic peptides, and echocardiographic signs of left ventricular dysfunction and was confirmed to have myocarditis. He had no history of cardiovascular disease, coronary artery disease, heart failure, lung disease, cancer, hypertension, or smoking [104].

Evaluation of cardiac manifestations in all COVID-19 confirmed or suspected cases is recommended to include but not limited to cardiac symptoms, chest X-ray or CT, unstable vital signs, cardiac biomarkers (ULN of CK-MB, Tn-I, Tn-T), electrocardiography, and echocardiography. It has been shown that cardiovascular complications in COVID-19 infection indicate a worse prognosis [4]. Early investigation, monitoring, and treatment are warranted. Physician recognition that viral myocarditis and notably COVID-19 simulating a myocardial infarction presentation with the frequent rise in troponin is integral to the diagnostic and treatment approach considered in COVID-19-infected patients [12]. Further research is needed to determine the time of onset of cardiac complications to make a recommendation on when this monitoring should be started.

#### 2.6.3. Does the Risk of Cardiovascular Disease Persist after COVID-19 Infection?

A cohort of 100 COVID-19-recovered patients showed independent of preexisting conditions, CMR evidence of cardiac involvement in 78%, and ongoing myocardial inflammation in 60% of patients 2 to 3 months after diagnosis [108]. Another study showed that in patients with no comorbidities, the death rate was 61.5% with elevated hs-Tnl and cardiac involvement [69]. Abnormalities included raised myocardial native T1 and native T2, myocardial late gadolinium enhancement (LGE) indicative of regional scarring, pericardial enhancement, elevated troponin, and decreased left ventricular ejection fraction [108]. This study suggests long-lasting cardiac involvement and exposes the need for ongoing investigation and monitoring of long-term cardiovascular complications from COVID-19.

While often bacterial, an increased risk of cardiovascular disease and complications has been linked to pneumonia in a 10-year follow-up study [109]. It is likely that COVID-19 as a primarily respiratory illness, whether pneumonia-like or respiratory distress, will cause similar adverse outcomes. A research suggested that COVID-19-recovered patients will continue to have subclinical or apparent clinical cardiovascular abnormalities, and that those with recovered cardiac function may continue to be at higher risk of developing cardiomyopathy and arrhythmia. The authors suggested developing cardiac screening tools for COVID-19-recovered patients now so that future complications can be caught and managed early. Myocardial injury, microinfarction, and fibrosis can lead to long-term cardiovascular sequelae postinfection such as cardiomyopathy, systolic and diastolic dysfunction, PVCs and VT, and atrial fibrillation [110]. The concern of myocardial inflammation led to investigating the use of cardiac magnetic resonance (CMR) imaging in competitive athletes recovered from COVID-19 [111]. CMR may allow risk stratification before the post-COVID patients can resume their athletic physical activity [111]. It is too early to know if long-term heart damage will be seen in these patients. Follow-up studies such as CISCO-19 [112] and other long-term screening measures are needed. Long-term follow-up studies of these diseases and their association with cardiovascular disease development are scarce. Follow-up studies of COVID-19 survivors are needed.

## 3. Conclusion

Acute cardiac injury is a common cardiovascular complication of COVID-19, and it occurs from direct myocardial injury, inflammation, myocardial oxygen supply, and demand mismatch, acute coronary events, and can be iatrogenic or from other unknown causes. Acute coronary events do not appear to be well documented but could result from plaque rupture or aggravation of the preexisting coronary disease. Heart failure can also result from myocardial injury or dysfunction or increased metabolic demand due to systemic disease, causing an acute decompensation of preexisting heart failure. Arrhythmia seems to occur in both mild and severe cases. However, little is known about the clinical value of these complications and manifestations of heart disease in COVID-19. Biomarker elevation, cardiac injury, and other cardiac sequelae may reflect the systemic disease and clinical course of COVID-19 infection.

Prolonged PT and aPTT, elevated d-dimer levels, and increased fibrin degradation levels are linked to a higher risk of mortality in COVID-19 patients. The mentioned lab values, excluding the PT/aPTT, signify a hypercoagulable state in a patient, which indicates potential thromboembolism. VTE has frequently been reported, and, upon presentation, patients with suspected and confirmed COVID-19 infection should be assessed for VTE risk. In treating patients with COVID-19, antiplatelet and anticoagulation therapies should be considered. Continued monitoring for signs of cardiac damage and coagulation (D-dimer) can help predict and potentially prevent COVID-19 complications.

The cardiovascular system is involved early in the disease course reflected in the release of prognostic and highly sensitive troponin and natriuretic peptides. Still, the actual time of onset of cardiovascular complications and symptoms has not been formally evaluated, leaving room for improvement in future research. Developing a cardiovascular screening protocol for COVID-19-recovered patients is crucial for monitoring the patients on a long-term basis by the primary care physician.

## Figures and Tables

**Table 1:** Cardiac complications.

	Eiros et al. [7]	Richardson et al. [113]	Bhatraju et al. [114]	Shi et al. [57]	Chen et al. [89]	Li et al. [25]	Wang et al. [115]	Liu et al. [11]	Ruan et al. [56]	Guan et al. [116]	Arentz et al. [75]	Huang et al. [117]	Wang et al. [13]	Yang et al. [110]	Zhou et al. [74]	Guo et al. [27]	Grimaud et al. [118]	Stefanini et al. [119]
*N*	139	5700	24	416	274	1527	339	137	150	1099	21	41	138	52	191	187	20	28
Age	52 (median)	63 (median)	64 (median)	64 (median)	62 (median)	n/a	71 (mean)	57 (median)		47 (median)	n/a	49 (median)	56 (median)	60 (mean)	56	59 (mean)	10 (mean)	n/a
Cardiac symptoms																		
Chest pain	29%	—	—	—	—	—	—	—	—	—	—	—	—	—	—	—	—	—
Palpitations	32%	—	—	—	—	—	—	—	—	—	—	—	—	—	—	—	—	—
Clinical findings									—									
CMR abnormalities	75%	—	—	—	—	—	—	—	—	—	—	—	—	—	—	—	—	—
ECG abnormalities	50%	—	—	—	—	—	—	—	—	—	—	—	—	—	—	—	—	—
Cardiomyopathy	—	—	—	—	—	—	—	—	—	—	33%	—	—	—	—	—	—	—
Myocarditis	37%	—	—	—	—	—	—	—	—	—	—	—	—	—	—	—	100%	—
Myocardial infarction	—	—	—	—	—	—	—	—	—	—	—	—	—	—	—	—	—	100%
Cardiac injury biomarkers	—	—	—	20%	44%	8-12%	21%	—	40% (*n* = 68)	—	—	12%	7%	23%	17%	28%	—	—
Elevated troponin	—	23%	15%	20%	41%	—	—	—	—	—	—	12%	—	—	17%	28%	—	—
Arrhythmia	—	—	—	—		—	10%	—	—		—	—	17%	—	—	6%	100%	—
Heart failure	—	—	—	—	24%	—	17%	—	—		—	—	—	—	23%	—		—
Shock	—	—	—	—		—	2%	—	—	1%	—	7%	9%	—	—	—		—
Coagulopathy	—	—	—	—		—		—	—		—	—	—	—	19%	34%		—
Medications																		
Antibiotic use	41%	—	—	—	91%	—	—	—	95%	—	—	—	18%	—	—	98%	—	—
Antiviral use	12%	—	—	—	86%	—	—	—	59%	—	—	—	90%	—	—	89%	—	—
Mechanical ventilation	—	12%	75%	—	43%	—	—	—	17%	2%	71%	10%	12%	42%	17%	24%	40%	—
ICU	—	23%	100%	—	—	—	—	—	27%	5%	100%	32%	26%	100%	26%	—	100%	4%
Non-ICU	—	77%	0%	—	—	—	—	—	73%	95%	0%	68%	74%	0%	74%	—	0%	96%
Comorbidities																		
Hypertension	12%	57%	—	31%	34%	17%	41%	10%	—	15%	—	15%	31%	—	30%	33%	—	71%
CV disease	6%	—	—	—	—	—	14%	7%	—	—	—	15%	15%	10%	—	—	—	—
Dyslipidemia	19%	—	—	—	—	—	—	—	—	—	—	—	—	—	—	—	—	—
Coronary artery disease	—	11%	—	11%	8%	—	—	—	—	3%	—	—	—	—	8%	11%	—	—
CHF	—	7%	—	4%	—	—	—	—	—	—	—	—	—	—	—	—	—	—
Cardiomyopathy	—	—	—	—	—	—	—	—	—	—	—	—	—	—	—	4%	—	—
Previous MI	—	—	—	—	—	—	—	—	—	—	—	—	—	—	—	—	—	11%
Survived	—	5147	12	359	161	—	274	121	82	1084	10	35	132	20	137	144	20	17
Death	—	553	12	57	113	—	65	16	68	15	11	6	6	32	54	43	0	11

**Table 2:** Definition and incidence of cardiac injury from COVID-19 studies.

Study	Patients (*N*)	Cardiac injury definition	Incidence of cardiac injury in COVID-19 infection (*N* (%))
Guo et al. [27]	187	Elevated troponin-T	52 (27.8%)
Huang et al. [117]	41	Elevated hs-troponin-I	5 (12%)
		ECG	
		Echo	
Wang et al. [13]	138	Elevated troponin-I	10 (7.2%)
		ECG	
		Echo	
Yang et al. [120]	52	Elevated hs-troponin-I	12 (23%)
Zhou et al. [74]	191	Elevated troponin-I	33 (17%)
		ECG	
		Echo	

**Table 3:** Vascular complications and pathology.

Study	Total population	Hearts studied	Mean/range age (years)	Cardiac comorbidities	Mean time to death	Cardiac enlargement	Other findings	Cardiac interstitial fibrosis	Epicardial mononuclear infiltrate	Myocardial infarction	Elevated D-dimer	Neurological vascular lesions	Venous thromboembolism	Other
Bryce et al. [93]	67	25	69 (range 34-94)	Hypertension (62.7%), coronary artery disease (31.3%), heart failure (14.9%), atrial fibrillation (13.4%)	9.5 days (range 0-61) from admission	Left ventricular hypertrophy (100%)	Myocyte hypertrophy and interstitial fibrosis	100%	60%	—	80.6%	Cerebral infarct (30%)	6%	Intravascular fibrin thrombi (25%), CD61 platelet aggregates or thrombi (31%), large pulmonary emboli (6%)
Fox et al. [95]	10 (African American)	9	Range 44-78	Hypertension (70%), atrial fibrillation (10%), heart failure (10%)	8.2 days (range 0-25) from admission; 11.6 days from symptom onset	Right ventricular dilation	Scattered myocyte necrosis; no significant lymphocytic infiltration	—	—	—	90%	—	—	Small, firm thrombi in peripheral parenchyma (100%)
Tian et al. [121]	4	2	Range 59-81	Hypertension (25%)	15-52 days from disease onset	—	—	100%	—	—	—	—	—	
Rapkiewicz et al. [100]	7	4	Range 44-65	Hypertension (85%), coronary artery disease (14%)	12.9 days (range 3-25) from symptom onset; mean 4.4 days from admission	—	Megakaryocytes with fibrin microthrombi in cardiac microvasculature (7/7)	—	25%	25%	100%	—	—	Pulmonary arterial thrombi (57%), venous thrombosis (29%), CD61 platelet aggregates or thrombi (100%)
Bradley et al. [96]	12	12	70.4	—	7 days (range 1-14) from symptom onset	—	Myocyte hypertrophy (12/12)	83%	—	—	—	—	—	Subsegmental pulmonary emboli (17%)
Giacca et al. [102]	41	30	Male -77; female -84	—	—	—	—	—	—	Cardiomyocyte damage suggestive of hypoxic injury (54%)	24%	—	—	Pulmonary thrombosis (77%)
Edler et al. [97]	80	80	Mean 79.2	Cardiomyopathy (11.25%), arrhythmia (1.25%), cardiac insufficiency (38.75%), atrial fibrillation (18.75%), hypertension (31.25%)	—	—	—	—	—	—	—	—	40%	Pulmonary embolism (21%)
Lax et al. [94]	11	11	Mean 80.5 (range 66-91)	Hypertension (81.8%), coronary artery disease (27.27%)	8.55 days (range 4-18) from symptom onset	Biventricular hypertrophy (100%), biventricular dilation (91%)	—	90.90%	—	—	86%	—	—	Pulmonary arterial thrombosis
Wichmann et al. [98]	12	12	Mean 73 (range 52-87)	Coronary/ischemic heart disease (50%)	—	Biventricular hypertrophy (25%)	—	—	—	50%	71%	—	58%	Pulmonary embolism
